# Long-term effect of early postnatal overnutrition on insulin resistance and serum fatty acid profiles in male rats

**DOI:** 10.1186/s12944-015-0094-2

**Published:** 2015-08-26

**Authors:** Fei Bei, Jia Jia, Yi-Qun Jia, Jian-Hua Sun, Fei Liang, Zhong-Yi Yu, Wei Cai

**Affiliations:** Xin Hua Hospital, Shanghai Jiao Tong University School of Medicine, 1665 Kongjiang Road, Shanghai, 200092 China; Shanghai Key Laboratory of Pediatric Gastroenterology and Nutrition, 1665 Kongjiang Road, Shanghai, 200092 China; Shanghai Institute for Pediatric Research, Shanghai Jiao Tong University School of Medicine, 1665 Kongjiang Road, Shanghai, 200092 China; Shanghai Center for Bioformation Technology, 1278 Keyuan Road, Shanghai, 201203 China; Shanghai University of Traditional Chinese Medicine, 1200 Cailun Road, Shanghai, 201203 China; Department of Neonatology, Shanghai Children’s Medical Center, Shanghai Jiao Tong University School of Medicine, 1678 Dongfang Road, Shanghai, 200127 China; School of Public Health, Physiotherapy & Sports Science, University College Dublin, Belfield, Dublin, 4 Ireland

**Keywords:** Early overnutrition, Insulin resistance, Insulin receptor substrate 1, Glucose transporter 4, Fatty acid

## Abstract

**Background:**

Increasing evidence suggests that overnutrition during the early postnatal period, a critical window of development, increases the risk of adult-onset obesity and insulin resistance. In this study, we investigated the impact of overnutrition during the suckling period on body weight, serum biochemistry and serum fatty acid metabolomics in male rats.

**Methods:**

Rats raised in small litters (SL, 3 pups/dam) and normal litters (NL, 10 pups/dam) were used to model early postnatal overnutrition and control, respectively. Serum glucose, triglyceride, high-density lipoprotein-cholesterol, free fatty acid, insulin and leptin concentrations were assayed using standard biochemical techniques. Serum fatty acids were identified and quantified using a gas chromatography–mass spectrometry-based metabolomic approach. mRNA and protein levels of key components of the insulin receptor signaling pathway were measured in epididymal fat and gastrocnemius muscle by quantitative PCR and western blotting.

**Results:**

SL rats were 37.3 % and 15.1 % heavier than NL rats at weaning and 16-weeks-old, respectively. They had increased visceral fat mass, adult-onset insulin resistance and glucose intolerance as well as elevated serum levels of free fatty acids and triglycerides. All detectable fatty acids were elevated in the serum of SL pups at weaning compared to NL controls, and significant increases in the levels of four fatty acids (palmitic acid, palmitoleic acid, oleic acid and arachidonic acid) persisted into adulthood. Moreover, a significantly positive correlation was identified between an insulin resistance index (HOMA-IR) and concentrations of myristic, palmitic, palmitoleic and oleic acid in serum at postnatal 16 weeks. Early postnatal overnutrition also resulted in a significant downregulation of insulin receptor substrate-1 (Irs-1), protein kinase B (Akt2) and glucose transporter 4 (Glut4) at the protein level in epididymal fat of SL rats at 16 weeks, accompanied by decreased mRNA levels for *Irs-1* and *Glut4*. In gastrocnemius muscle, *Akt2* and *Glut4* mRNA and Glut4 protein levels were significantly decreased in SL rats.

**Conclusions:**

This study demonstrates that early postnatal overnutrition can have long-lasting effects on body weight and serum fatty acid profiles and can lead to impaired insulin signaling pathway in visceral white adipose tissue and skeletal muscle, which may play a major role in IR.

## Introduction

The dramatic increase in the prevalence of obesity in recent years is a global health problem because it significantly increases the risk of adult-onset metabolic diseases such as glucose intolerance, insulin resistance (IR), Type 2 diabetes and cardiovascular disorders. In China, the overall prevalence of diabetes and prediabetes in adults has been estimated at 113.9 million and 493.4 million, respectively, in 2013 [1]. Insulin resistance (IR) plays a pivotal role in the development of metabolic diseases [2]. In addition to causing the excessive energy intake in developed countries, it has been recognized that nutrient availability during early postnatal life has an important influence on obesity, IR and resulting adult health [3–5]. In rodents, early postnatal overnutrition can be induced by a reduction of the number of pups per dam from 10–12 (normal litter, NL) to 3–4 (small litter, SL), resulting an increased intake of calories and fat. Rats or mice raised in SL develop a “metabolic-syndrome-like” phenotype in adulthood including overweight, obesity, IR, hyperinsulinemia and heperleptinemia [6–8]. The SL model has thus been widely used to investigate the long-term consequences of neonatal overnutrition and the molecular mechanisms underlying these events.

Insulin plays a primary role in the regulation of total body glucose homeostasis by increasing glucose uptake in muscle and adipose tissue, and inhibiting glucose production in the liver. Insulin-dependent glucose uptake occurs in skeletal muscle and adipose tissue via the insulin receptor signaling pathway. Insulin binding to the α-subunit of the insulin receptor leads to phosphorylation of insulin receptor substrate (IRS) proteins and the subsequent activation of phosphatidyl 3-kinase/protein kinase B (Akt2), which results in translocation of the glucose transporter 4 (GLUT4) from storage vesicles to the plasma membrane and stimulation of glucose uptake [9]. Decreased expression of any of the main components of this pathway such as IRS, Akt2 and GLUT4 may contribute to glucose intolerance, IR and type 2 diabetes. In particular, GLUT4 in muscle and adipose tissue is essential for normal global glucose homeostasis. Studies have demonstrated that heterozygous *GLUT4*+/− mice which exhibit decreased GLUT4 protein in muscle and adipose tissue display IR and a tendency toward diabetes that is consistent with a major role of GLUT4 in glucose disposal [10–12].

Studies have suggested that increased circulating concentrations of individual fatty acids may be implicated in causing IR in humans [13–15]. Using metabolomics, a promising method of identifying biomarkers of short- and long-term physiological or pathological changes in the organism [16], a number of specific lipids have been suggested as potential biomarkers for IR in humans. Some of these are involved in causing IR by interfering with the insulin receptor signaling pathway [14, 17]. In this study, we employed a metabolomic approach to identify and quantify individual fatty acids in the serum of SL and NL rats both at weaning and during adulthood. We further examined the expressions of main components of the insulin receptor signaling pathway in visceral adipose tissues and skeletal muscle of adult rats to determine whether blunting of the insulin signaling pathway in these tissues may also contribute to IR.

## Results

### Effect of early postnatal overnutrition on body weight during development

**Fig. 1:**
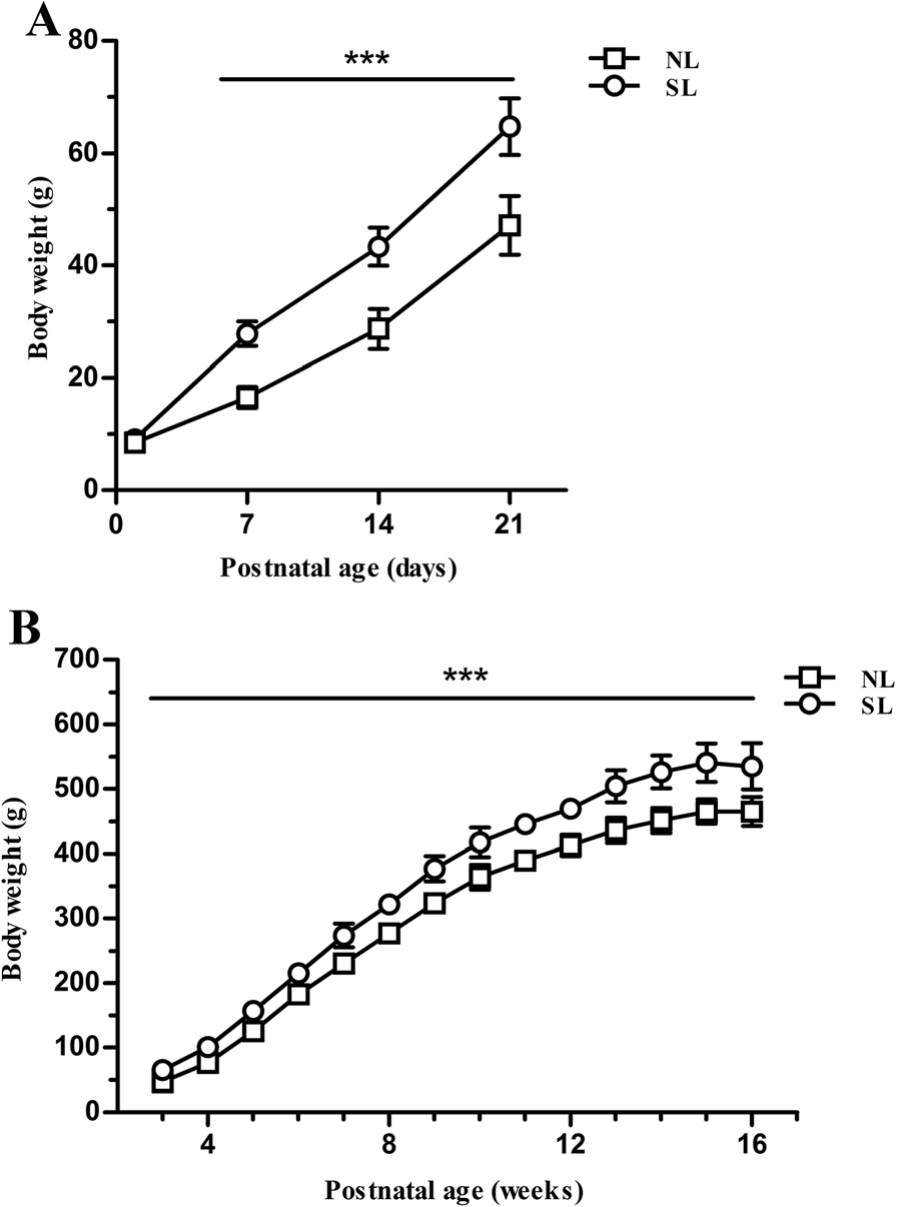
Body weights of rats during the first 16 weeks of life. Body weight growth curves are shown for rats from normal litters (NL, □) and small litters (SL, ○) during suckling period (**a**) (*n* = 24, 8 litters from each group) and postweaning period (**b**). (*n* = 10–12, 4 litters from each group) Results are expressed as mean ± standard error of mean (SEM). Age, litter size, and the interaction were significant, ^***^ *P <* 0.001

To investigate the effect of postnatal overnutrition on body weight, we adjusted litter sizes on postnatal day 2 to either ten male pups/dam (normal litter, NL) or three male pups/dam (small litter, SL) and monitored body weight over the first 16 weeks of life. NL and SL pups had the same average body weight at the time of litter adjustment, but SL pups gained weight more rapidly and were already significantly heavier at postnatal day 7 (Fig. 1a). At weaning (postnatal day 21), SL pups were 37.3 % heavier than NL pups (64.8 g *vs.* 47.2 g), and this significant difference in body weight between the two groups persisted into adulthood (Fig. 1b). At the age of 16 weeks, SL rats were 15.1 % heavier than NL rats (534.8 g *vs.* 464.8 g).

### Food intake, organ weights and metabolic parameters in the NL and SL groups

To further characterize the effects of early postnatal overnutrition in adulthood, rats were killed at 16 weeks and their livers, hindlimb gastrocnemius muscles, epididymal fat pads and perirenal fat pads were harvested and weighed. Liver, gastrocnemius and epidiymal fat pads were all significantly heavier in rats raised in SL compared with NL; however after normalization for body weight only the epididymal fat pads were significantly heavier in the SL rats (Table 1). Furthermore, serum from 16-week-old SL rats was found to contain significantly higher levels of triglycerides and free fatty acids compared to NL rats. There were no differences in the serum leptin and the density lipoprotein-cholesterol (HDL-C) concentrations between the two groups (Table 2).

**Table 1:** Food intake, and body and organ weights in 16 week old rats

	NL(*n* = 10)	SL(*n* = 10)
Food intak (g/d)	28.67 ± 2.07	29.83 ± 2.32
Body weight (BW) (g)	464.8 ± 22.4	534.8 ± 35.8^***^
Organ weights		
Liver (g)	11.4 ± 0.8	13.0 ± 1.0^**^
Epididymal fat pad (g)	6.1 ± 1.0	8.5 ± 1.8^**^
Perirenal fat pad (g)	10.9 ± 2.9	13.0 ± 2.7
Gastrocnemius (g)	5.9 ± 0.4	6.4 ± 0.3^**^
Liver/BW (%)	2.33 ± 0.16	2.43 ± 0.12
Epididymal fat/BW (%)	1.24 ± 0.14	1.58 ± 0.28^*^
Perirenal fat/BW (%)	2.20 ± 0.48	2.42 ± 0.43
Gastrocnemius/BW (%)	1.20 ± 0.04	1.21 ± 0.07

Values are mean ± S.E.M. (n = 10, 4 litters from each group). NL: normal litter; SL: small litter, ^*^ *P <* 0.05, ^**^ *P <* 0.01, ^***^ *P <* 0.001

**Table 2:** Blood serum biochemistry in 16 week old rats from normal and small litters

	NL(*n* = 6)	SL(*n* = 6)
TG (mmol/L)	0.93 ± 0.26	1.39 ± 0.41^*^
HDL-C (mmol/L)	0.52 ± 0.09	0.51 ± 0.05
FFA (mmol/L)	0.34 ± 0.04	0.41 ± 0.05^**^
Leptin (pg/ml)	7.28 ± 1.61	8.3 ± 3.6

Values are mean ± S.E.M. (n = 6, 4 litters from each group) NL: normal litter; SL: small litter; TG: triglyceride; HDL-C: high density lipoprotein cholesterol; FFA: free fatty acid. ^*^ *P <* 0.05, ^**^ *P <* 0.01

### Serum levels of fasting glucose and insulin

**Fig. 2:**
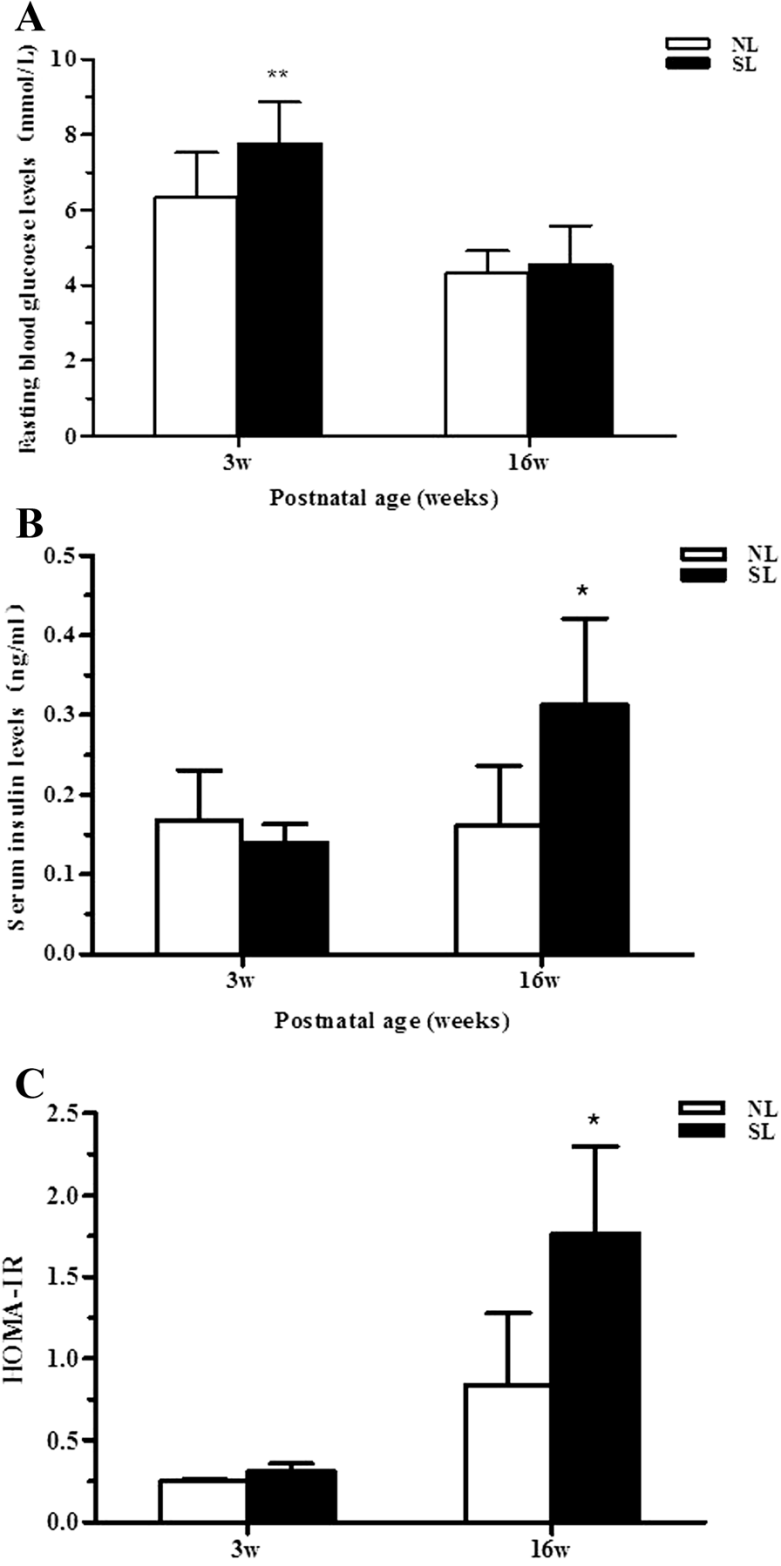
Levels of fasting serum glucose (**a**), insulin (**b**) and homeostasis model assessment-insulin resistance index (HOMA-IR) (**c**) in rats at 3 weeks (*n* = 8, 8 litters from each group) and 16 weeks(*n* = 5, 4 litters from each group) of age from normal litters (NL, open bars) and small litters (SL, closed bars). Values are expressed as mean ± S.E.M.. ^*^ *P <* 0.05, ^**^ *P <* 0.01

A commonly used estimate of insulin resistance is the homeostasis model assessment-insulin resistance index (HOMA-IR), which is proportional to the product of serum glucose and insulin levels in fasted animals. We found that glucose levels were significantly higher in the serum of fasted SL pups than NL pups at 3 weeks of age, but the groups did not differ after weaning (Fig. 2a). By contrast, significantly elevated serum insulin concentrations were observed in SL rats at 16 weeks of age, but not before (Fig. 2b). At 16 weeks the HOMA-IR index was significantly higher in SL rats compared with NL rats, indicating the development of insulin resistance (Fig. 2c).

### Early postnatal overnutrition induces glucose intolerance

**Fig. 3:**
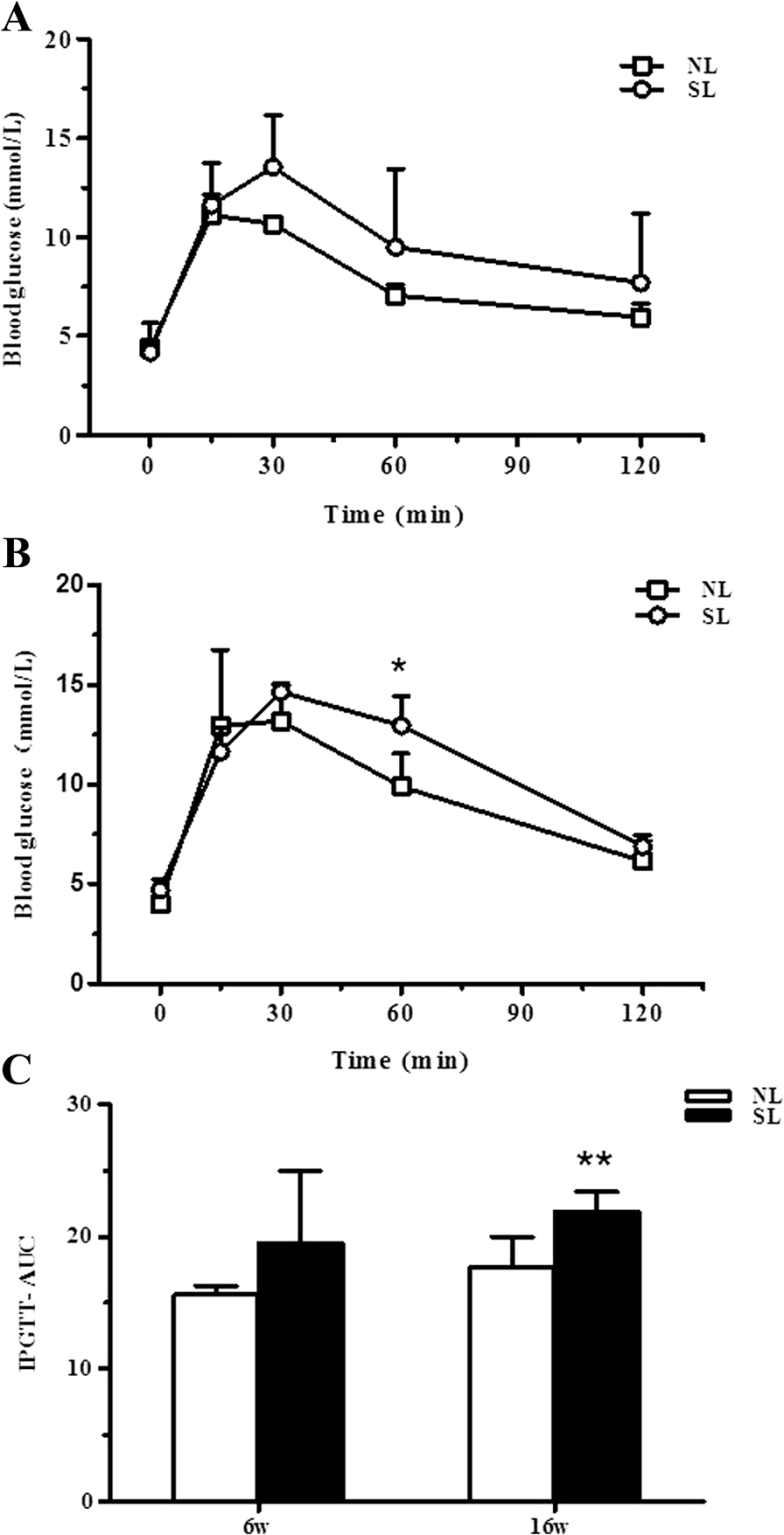
Glucose tolerance in rats aged 6 and 14 weeks. Intraperitoneal glucose tolerance test (IPGTT) at 6 weeks (**a**) and 14 weeks (**b**) in rats from normal litters (NL, □) and small litters (SL, ○). Comparison of the area under the curve (AUC) (**c**) between NL (open bar) and SL (closed bar) rats at each time point. Results are expressed as mean ± S.E.M. (*n* = 5, 4 litters from each group). ^*^ *P <* 0.05, ^**^ *P <* 0.01

To further investigate insulin resistance in SL and NL rats, we performed an intraperitoneal glucose tolerance test (IPGTT) at 6 and 14 weeks of age. Rats were given an intraperitoneal glucose load, then blood glucose levels were measured at regular intervals to monitor their return to baseline over a two hour period. At 6 weeks of age, there was no difference in blood glucose levels between the two groups at any timepoint (Fig. 3a). However, at 14 weeks of age, SL rats had significantly higher serum glucose levels at 60 min and a significantly increased area under the curve (AUC) when compared to NL rats (Fig. 3b, c), indicating that early postnatal overnutrition induces glucose intolerance in adult rats. The glucose intolerance observed in adult SL rats indicates an imbalance between insulin production and action in these animals due to decreased insulin secretion from pancreatic island β cells and/or decreased insulin sensitivity in target organs.

### Effect of early postnatal overnutrition on mRNA expression and protein levels for components of insulin signaling pathway from skeletal muscle and visceral adipose tissue

**Fig. 4:**
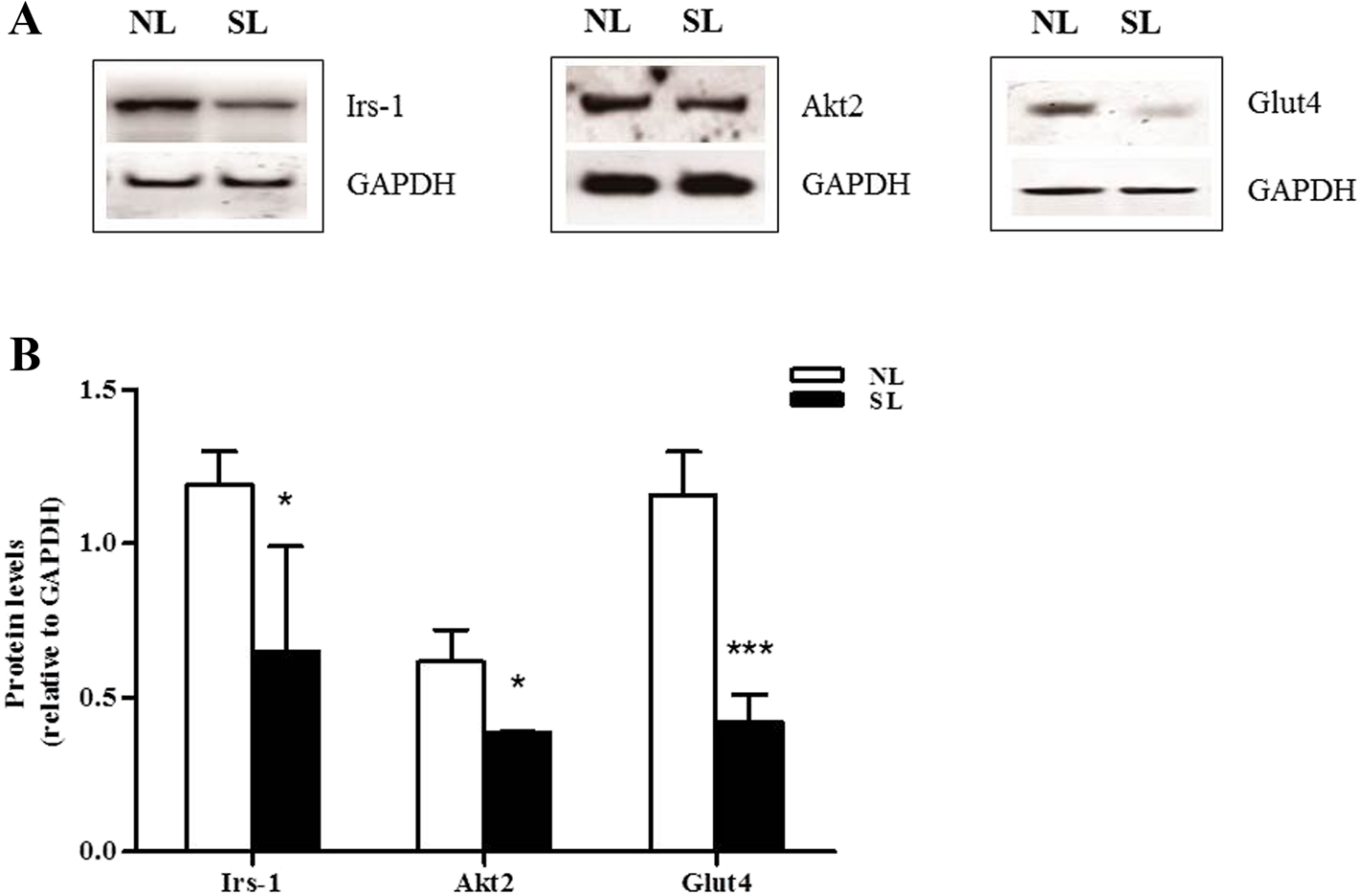
Protein expression of key insulin signaling components in epididymal fat at 16 weeks. Protein levels of insulin receptor substrate-1 (Irs-1), protein kinase B (Akt2) and glucose transporter 4 (Glut4) in the epididymal fat of rats from normal litters (NL) and small litters (SL) were assessed by western blotting. Representative blots are shown in (**a**). Relative protein levels were quantified by scanning densitometry of the bands (**b**). Results are expressed as mean ± S.E.M (n = 6, 4 litters from each group). ^**^ *P <* 0.01, ^***^ *P <* 0.001

**Fig. 5:**
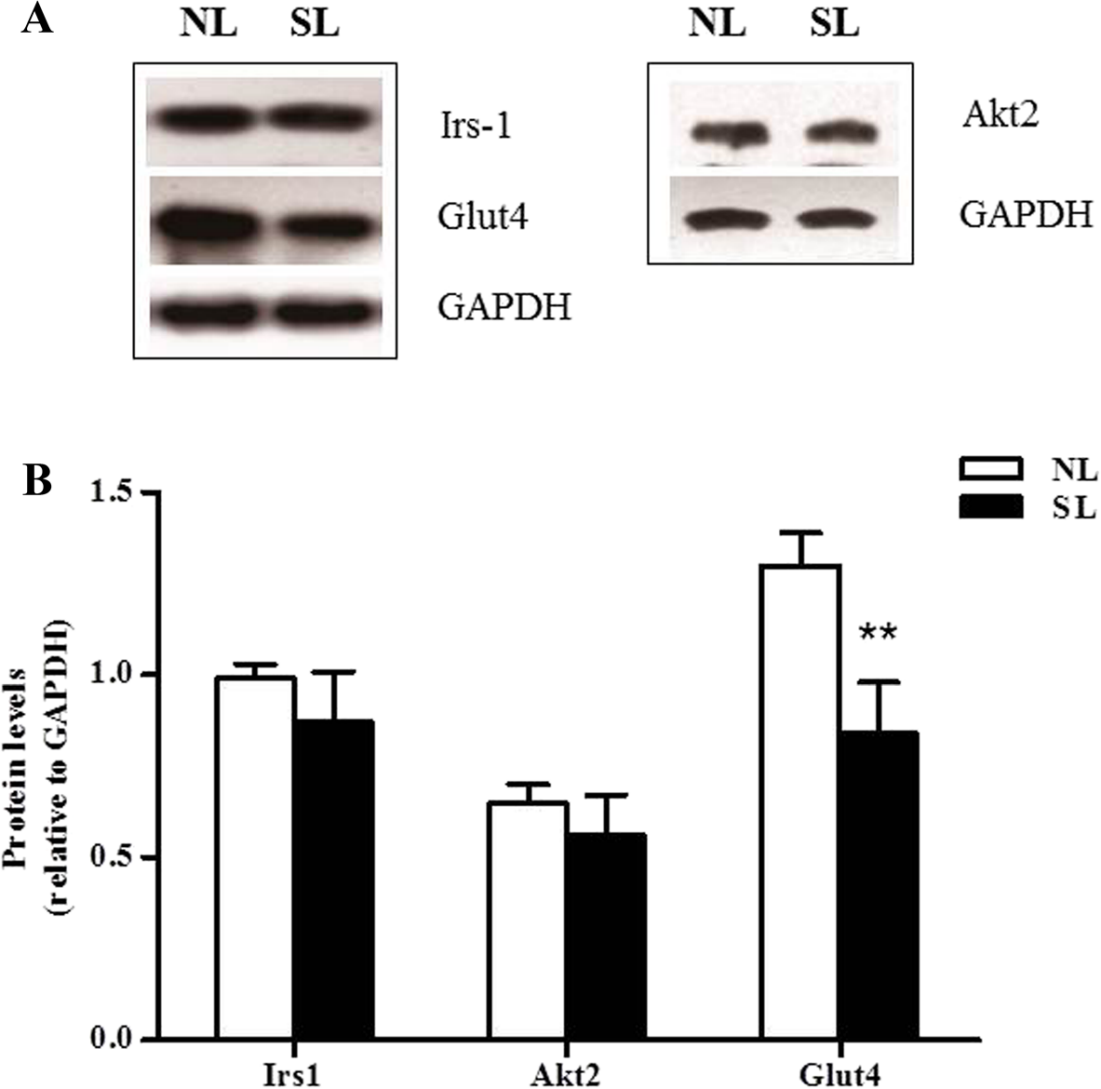
Protein expression of key insulin signaling components in the gastrocnemius muscle at 16 weeks. Protein levels of Irs-1, Akt2 and Glut4 in the gastrocnemius of rats from NL and SL were assessed by western blotting. Representative blots are shown in (**a**). Relative protein levels were quantified by scanning densitometry of the bands (**b**). Results are expressed as mean ± S.E.M (*n* = 6, 4 litters from each group). ^**^ *P <* 0.01

Insulin resistance may arise from downregulation of key components of the insulin signaling pathway. To investigate whether this was the case following postnatal overnutrition, the levels of three such proteins, Irs-1, Akt2 and Glut4, were measured by western blotting in lysates from epididymal fat and gastrocnemius muscle isolated from SL and NL rats at 16 weeks of age. A significant decrease in expression of all of the above proteins was observed in the epididymal fat of SL rats compared to NL controls (*P* < 0.05) (Fig. 4). In skeletal muscle, the protein content of Glut4 was found to be significantly decreased in SL rats compared to NL, but no significant differences were observed in the protein levels of Irs-1 and Akt2 between the two groups (Fig. 5).

**Fig. 6:**
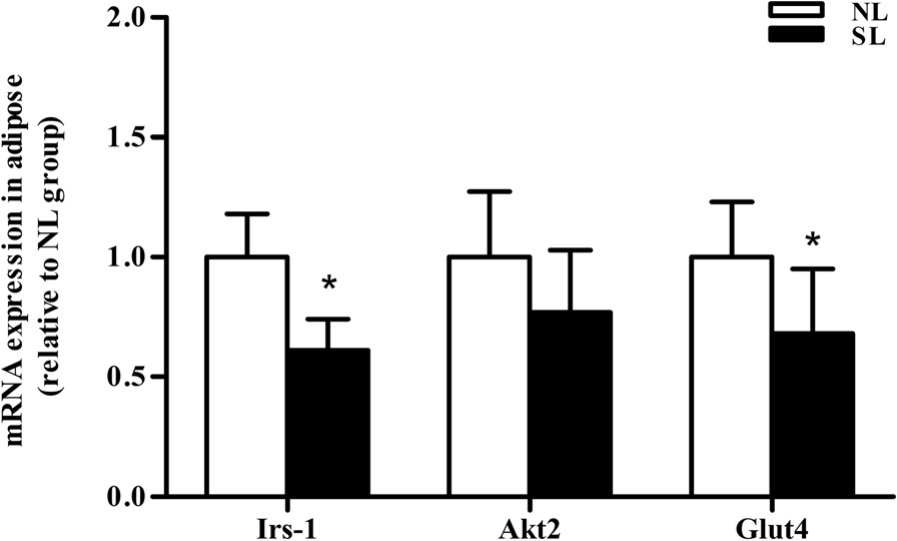
mRNA expression of key insulin signaling components in epididymal fat at 16 weeks. mRNA level of *Irs-1*, *Akt2* and *Glut4* were assessed by quantitative PCR in the epididymal fat of rats from normal litter (NL, open bar) and small litter (SL, closed bar). Results are expressed as mean ± S.E.M (*n* = 6, 4 litters from each group). ^*^ *P <* 0.05

**Fig. 7:**
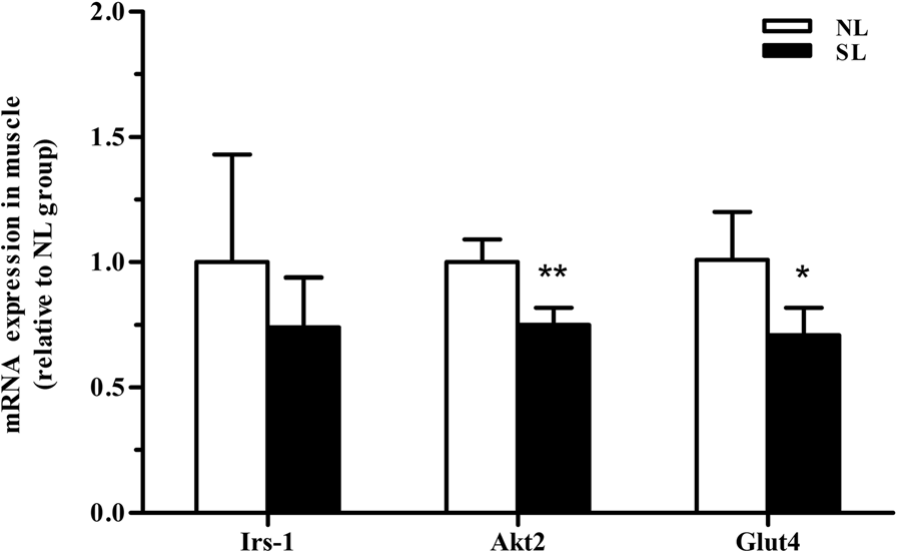
mRNA expression of key insulin signaling components in the gastrocnemius muscle at 16 weeks. mRNA levels of *Irs-1, Akt2 and Glut4* were assessed by quantitative PCR in the gastrocnemius of rats from normal litters (NL, open bars) and small litters (SL, closed bars). Results are expressed as mean ± S.E.M (*n* = 6, 4 litters from each group). ^*^ *P <* 0.05, ^**^ *P <* 0.01

In order to evaluate whether the changes in protein content are associated with changes in the levels of mRNA, quantitative real-time PCR was performed on mRNA isolated from adult SL and NL rats. At 16 weeks of age, early postnatal overnutrition resulted in a significant decrease in mRNA levels of *Irs-1* and *Glut4* (*P* < 0.05) in epididymal fat, paralleling the results of protein measurement (Fig. 6). In muscle a significant decrease in *Glut4* mRNA (*P* < 0.05) was observed (Fig. 7), consistent with the western blot data, in addition to a marked reduction in *Akt2* mRNA expression (*P* < 0.05, Fig. 7) which did not reflect a significant change at the protein level. In contrast, the protein level of Akt2 in epididymal fat was significantly lower in SL rats compared to NL rats, unlike the mRNA expression levels (*P* > 0.05) (Fig. 6). In line with the protein comparison, there is no significant difference in *Irs-1* mRNA expression between two groups in skeletal muscle (Fig. 7).

### Effect of early postnatal overnutrition on serum fatty acid metabolomics

Numerous studies have shown that obesity induces an increase in circulating free fatty acids (FFAs), and this is considered to play an important role in the development of IR [18–21]. We therefore employed a metabolomics approach to investigate serum fatty acids profiles in rats at weaning (day 21) and at 16 weeks of age. Using gas chromatography–mass spectrometry (GC-MS), 11 fatty acids were detected in serum at weaning and 10 fatty acids were detected at 16 weeks of age. These fatty acids included saturated fatty acids (SFAs) (C12:0, C14:0, C15:0, C16:0 and C18:0), monounsaturated fatty acids (MUFAs) (C16:1 and C18:1), and polyunsaturated fatty acids (PUFAs) (C18:2n-6, C18:3n-3, C20:4n-6 and C20:5n-3). Lauric acid (C12:0) was identified in the serum of rats at weaning but not at 16 weeks. All the fatty acids identified except lauric acid (C12:0) are long chain fatty acids. Palmitic acid (C16:0), stearic acid (C18:0), oleic acid (C18:1), linoleic acid (C18:2n-6) and arachidonic acid (C20:4n-6) accounted for the majority of all fatty acids (Table 3).

**Table 3:** Serum fatty acid profiles in 3 and 16 weeks old rats

	3 wk		16 wk	
	NL(*n* = 5)	SL(*n* = 5)	NL(*n* = 6)	SL(*n* = 6)
C12:0	3.19 ± 1.03	16.92 ± 9.74^*^	-	-
C14:0	15.33 ± 5.09	34.8 ± 12.09^*^	1.86 ± 0.56	2.51 ± 0.55
C15:0	2.15 ± 0.25	3.1 ± 0.27^*^	1.92 ± 0.73	1.9 ± 0.91
C16:0	205.66 ± 32.11	313.67 ± 70.04^*^	174.19 ± 46.54	241.28 ± 24.38^*^
C16:1	2.91 ± 0.64	8.64 ± 1.34^***^	5.86 ± 2.13	9.55 ± 3.14^*^
C18:0	85.94 ± 13.6	138.86 ± 31.22^**^	93.08 ± 21.44	122.06 ± 23.42
C18:1	78.06 ± 15.52	113.24 ± 16.56^*^	56.27 ± 18.42	88.71 ± 14.69^*^
C18:2n6	114.39 ± 29.43	173.90 ± 40.52^*^	110.02 ± 40.26	145.95 ± 13.11
C18:3n3	2.74 ± 1.69	3.44 ± 0.75	1.73 ± 1.05	2.32 ± 1.19
C20:4n6	154.57 ± 26.42	291.87 ± 72.56^**^	176.61 ± 56.01	245.59 ± 53.87^*^
C20:5n3	7.86 ± 2.35	14.92 ± 1.83^**^	5.07 ± 1.68	5.68 ± 2.28
SFAs	312.26 ± 50.3	507.34 ± 122.25^**^	271.04 ± 67.86	367.86 ± 46.39^*^
MUFAs	80.97 ± 15.56	121.87 ± 17.71^**^	62.12 ± 19.76	98.25 ± 14.62^**^
n-6PUFAs	268.96 ± 52.33	465.77 ± 109.44^**^	286.63 ± 94.05	391.54 ± 60.78
n-3PUFAs	10.05 ± 4.17	18.37 ± 1.17^*^	6.45 ± 1.92	8 ± 3.16

Values are mean concentration (μg/ml) ± SEM (3 w, n = 5, 8 litters from each group; 16 w, n = 6, 4 litters from each group). NL: normal litter; SL: small litter; SFA: saturated fatty acid; MUFA: monounsaturated fatty acid; PUFA: polyunsaturated fatty acid. ^*^ *P <* 0.05, ^**^ *P <* 0.01, ^***^ *P <* 0.001

At 3 weeks of age, a significant increase (*P <* 0.05) was observed in the serum concentration of all fatty acids except for C18:3n3 in SL rats compared to NL rats (Table 3). This elevation in the overall level of serum fatty acids in the SL group may be attributed to the high fat content of milk and the increased calorie intake of these rats during early postnatal life. At 16 weeks, serum levels of several of the identified fatty acids were no longer significantly different between the two groups; however, significant increases in serum levels of C16:0, C16:1, C18:1 and C20:4n-6 (and the consequent elevation in total SFA and MUFA levels) in SL rats compared to NL persisted into adulthood (*P <* 0.05) (Table 3). To determine the relationship between serum fatty acid levels and insulin resistance (estimated by the HOMA-IR index) in adulthood, Pearson’s correlation coefficients were calculated for each individual fatty acid at 16 weeks. This analysis revealed significantly positive correlations for myristic acid (C14:0), palmitic acid (C16:0), palmitoleic acid (C16:1) and oleic acid (C18:1) (Table 4).

**Table 4:** Correlation between serum fatty acid concentrations and insulin resistance index (HOMA-IR) at 16 weeks

Fatty acid	*R*	*P*-value
C14:0	0.798^**^	0.003
C15:0	0.178	0.601
C16:0	0.614^*^	0.044
C16:1	0.745^**^	0.008
C18:0	0.382	0.247
C18:1	0.674^*^	0.023
C18:2n6	0.445	0.170
C18:3n3	0.261	0.467
C20:4n6	0.264	0.433
C20:5n3	0.291	0.385

*R* indicates Pearson’s correlation co-efficient calculated between serum fatty acid levels and the homeostatic model assessment-insulin resistance (HOMA-IR) index in NL and SL groups at 16 weeks of age. ^*^ *P <* 0.05, ^**^ *P <* 0.01

## Discussion

Overnutrition in early postnatal life, a critical window for development, can give rise to long-term consequences for body weight and metabolic diseases. Breast fed infants, compared to those given formula feeds, have lower daily calorie intakes and a 40 % decreased risk of developing type 2 diabetes [22, 23]. Some studies have shown that formula feeding is a risk factor for later overweight [24, 25]. Quantitative and qualitative overnutrition associated with formula feeding during infancy has potential risks for later metabolic health [26]. In rats, early postnatal overnutrition caused by manipulations to produce small litters has been shown to cause persistent overweight, hyperinsulinemia, hyperleptinemia and IR [8, 27, 28]. SL pups have also been subjected to quantitative and qualitative overnutrition during early postnatal life [26, 29]. This overweight/obsese phenotype has been extensively investigated in terms of hypothalamic mechanisms; however, molecular mechanisms underlying the increased susceptibility to IR in peripheral organs are poorly appreciated. To the best of our knowledge, no study has yet investigated the effect of early postnatal overnutrition on serum fatty acid metabolomics. In this study, we focused on the consequences of overnutrition during early postnatal life on serum fatty acid profiles and alterations of insulin signaling cascades in skeletal muscle and visceral white adipose tissue in order to determine whether early nutritional experience may influence the levels of key components of the insulin signaling pathway which play an important role in IR.

Consistent with previous studies [27, 28, 30, 31], our data show that early overnutrition leads to a persistent increase in body weight and adipose tissue accumulation, adult-onset hyperinsulinemia, and IR. During the glucose tolerance tests, SL rats exhibited higher glycaemia and AUC at 14 weeks than NL rats, suggesting decreased insulin sensitivity in this group. We found no difference between the groups in the serum leptin concentration at 16 weeks of age. This is consistent with previous findings [31–34]. Leptin is mainly produced by subcutaneous adipocytes [35]. Adult SL rats are reported to have a normal subcutaneous fat mass, but a higher visceral fat mass [33]. However, visceral adipocytes have a lower leptin content than subcutaneous adipocytes due to adipocyte dysfunction [31]. This observation may explain the unchanged serum leptin concentration, and resulting unchanged food intake at 16 weeks of age. Numerous mechanisms have been proposed to underlie IR in animals raised in small litters, including blunting of insulin signaling, increased levels of circulating fatty acid and some adipocytokines, increased oxidative stress, abnormal integration and projection of orexigenic and anorexigenic neurons, impairment of the hypothalamic-pituitary-adrenal glucocorticoid axis, and aberrant epigenetic modifications [8, 27, 36–39].

Previous studies have shown that early postnatal overfeeding increases serum levels of free fatty acids (FFA) [28, 40], a finding that we confirmed in our model. FFA released from adipose tissue into the circulation have been shown to attenuate glucose transport by impairing insulin signaling in skeletal muscle and adipose tissue, resulting in decreased glycogenesis in these tissues and increased serum glucose [20, 41]. In addition, high FFA levels contribute to increased glucose output in liver and aggravate hyperglycemia, eventually leading to IR. IR in turn results in inhibition of lipolysis [42], further increasing circulating FFA levels and causing a vicious cycle which can lead to type 2 diabetes mellitus.

The insulin receptor signaling pathway is the major mechanism by which insulin target tissues maintain their systemic and local insulin sensitivity. Decreased expression of Irs-1, Akt2 and Glut4, all key components of the insulin signaling pathway, results in reduced insulin action and IR. Our data showed that Irs-1, Akt2 and Glut4 levels dramatically decreased in epididymal fat of SL rats compared with NL rats, indicating that insulin signaling was severely impaired in visceral adipose tissue following overnutrition during early postnatal life. In gastrocnemius, mRNA levels of *Akt2* and *Glut4* and protein levels of Glut4 also decreased, suggesting the insulin signaling was also blunted in skeletal muscle. These data confirm and extend previous findings using the same small litter size model [43, 44]. Low cellular Irs-1 expression was previously demonstrated to be associated with low Glut4 expression and impaired insulin-stimulated glucose transport in IR [45]. It is clear that IRS-1 gene disruption in mice leads to a marked resistance to the glucose-lowering effects of insulin [46]. Likewise, Akt2 deficiency in mice is associated with IR and a diabetes mellitus-like syndrome [47]. Abel *et al.* reported that mice which exhibit an adipose-selective reduction of Glut4 developed IR in muscle and liver, despite having no impairment of Glut4 expression in these tissues [48]. Thus, adipose tissue may contribute more to global glucose homeostasis than is reflected by its glucose uptake, which accounts for only 5-10 % of the total glucose load [45, 49]. For rats raised in small litters, the combination of low expression of Glut4 in both adipose tissue and skeletal muscle will inevitably exacerbate adult-onset IR. Surprisingly, in the present study, we did not observe a correlation between Akt2 mRNA and protein levels. Various regulatory mechanisms at the level of transcription, post-transcription, translation or protein degradation may contribute to this variation of mRNA and protein concentrations [50].

Not only the levels, but also the composition of circulating fatty acids may have an impact on the development of IR. To investigate this, we employed metabolomic profiling to identify and measure the levels of individual fatty acids in serum at 3 weeks and 16 weeks of age. We observed a sustained increase in the levels of SFA palmitic acid (C16:0), MUFAs palmitoleic acid (C16:1) oleic acid (C18:1), and PUFA arachidonic acid (C20:4n-6) in adult SL rats. SFAs are thought to contribute to IR by several mechanisms, including the activation of c-Jun N-terminal kinase (JNK)/IκB kinase (IκK) signaling [21], inhibition of AKT or IRS phosphorylation by their metabolites [51, 52], and the direct inhibition of GLUT4 activation in insulin target organs [53]. The attenuated action of insulin on circulating glucose levels causes the pancreas to secrete more insulin to maintain blood glucose homeostasis, leading to hyperglycemia and IR. Palmitic acid, which comprises the overwhelming majority of long chain SFAs, has been widely used as an inducer of IR in hepatocytes [54], adipocytes [55] and skeletal muscle cells [56]. We detected a significantly positive correlation between IR index and serum myristic and palmitic acid concentrations which is supported by a study by Xu *et al.*, who reported that significantly raised myristic, palmitic and stearic acid levels in serum are associated with type 2 diabetes and increased fasting glucose levels using mass spectrometry-based metabolic profiling [57]. Palmitate not only directly interacts with the insulin receptor and IRS proteins via activation of protein kinase Cθ [58, 59], but also causes increased mitochondrial reactive oxygen species production, which is related to inhibition of insulin signaling in skeletal muscle cells [60]. Elevated palmitic acid in serum has furthermore been proposed as a diagnostic biomarker for IR [17, 61].

In this work, two MUFAs, palmitoleic acid (C16:1) and oleic acid (C18:1), were persistently and significantly elevated in the serum of SL rats. Both these fatty acids also positively correlated with IR index in adult rats, consistent with the fact that both have been previously suggested as diagnostic biomarkers for IR [17, 61]. An increased concentration of palmitoleate has been reported to be associated with both raised triglycerides levels and with IR [14]. A study by Kusunokiy and colleagues showed that the serum concentrations of palmitoleic acid and oleic acid were positively correlated with HOMA-IR in individuals with type 2 diabetes mellitus [15]. Oleic acid was shown to downregulate GLUT4 expression in skeletal muscle cells via NF-κB and sterol-regulatory-element-binding-protein-1 (SREBP1), which may participate in the fatty acid related pathophysiology of IR [62].

Compared with long chain SFAs and MUFAs, the exact effect of long chain PUFAs on IR are unknown. We detected two n-6 PUFAs in serum, linoleic acid (C18:2n-6) and arachidonic acid (AA, C20:4n-6), of which the latter was consistently elevated in SL rats compared with NL rats. Previous studies of the effect of AA on IR have been contradictory. Supplementation of AA was reported to prevent whole-body IR induced by a high-fat diet in rats, suggesting a protective effect [63]. However, as a precursor to inflammatory transmitters such as prostaglandins and leukotrienes, AA may aggravate IR via the inflammatory pathway. Moreover, Williams and colleagues identified a positive relationship between AA content in adipose tissue and metabolism dysregulation including abdominal obesity, hypertriglyceridemia and elevated fasting glucose in adult participants [64]. However, we found no evidence of a positive correlation between AA and HOMA-IR. The precise effect of increased serum concentration of AA in SL rats in the current work is unclear and further studies will be needed to confirm and complete our knowledge in this field, such as systemic and local evaluation of IR and inflammation after AA supplementation.

## Conclusions

Our data provide evidence that early postnatal overnutrition induced by small litters can lead to sustained increased body weight in the offspring, the accumulation of white visceral adipose tissue, elevated serum fatty acid and triglyceride levels, and increased fasting serum glucose in adult male rats. Decreased levels of the main components of the insulin receptor signaling pathway, Irs-1, Akt2 and Glut4, in white visceral adipose tissue and skeletal muscle may be responsible for adult-onset IR in SL rats. Ours is the first study to report that persistent high serum levels of SFA palmitic acid (C16:0), MUFAs palmitoleic acid (C16:1) and oleic acid (C18:1), due to nutrition overload during early postnatal life, may play a role in the development of IR in adulthood by interfering with the insulin receptor signaling pathway. However, it should be noted that the power of the current study to identify specific fatty acids as biomarkers for IR is limited by the fact that serum fatty acid profiles were only assessed at 3 and 16 weeks. In the future, a longitudinal study will be needed to provide more definitive evidence for the correlation between changes of fatty acid profile and adult-onset IR. Our findings strengthen the evidence for the potency and long-term effects of early postnatal overnutrion in reprogramming metabolic regulatory mechanisms. Avoiding overfeeding during early postnatal life appears to be an important and effective means of preventing adverse postnatal programming, and long term metabolic problems.

## Methods

### Animals and experimental design

This study was approved by the University Committee on the Use and Care of Animals at Shanghai University. Sprague–Dawley rats (Slaccas, Shanghai, China) were housed under controlled temperature conditions (23 ± 2 °C) and a 12 h light/dark cycle with *ad libitum* access to a standard rodent laboratory chow (14.44 kJ/g) and water. The mean size of birth litters used in the study was 10–12 pups/dam. At postnatal day 2, litters were assigned randomly to either NL or SL groups. For the SL group, litter size was adjusted to three male pups/dam. NL pups were raised in litters of 10 pups/dam, containing male and female pups. All the pups were nursed by their natural dams. The pups were weaned on postnatal day 21, and three male pups from each litter were seperated. After that, all male rats were housed three per cage and fed standard chow and water *ad libitum*. Only male rats were analyzed in the study.

### Serum and tissue collection

At 3 and 16 weeks, rats were anesthetized by an intraperitoneal injection of chloral hydrate (300 mg/kg body weight) after overnight fasting (12 h) and blood was obtained from the heart. The blood was collected in nonheparinized tubes and centrifuged (3100 g, 25 °C, 15 min). The separated serum was stored at −80 °C. Gastrocnemius, epididymal and perirenal fat pads were dissected, weighed and frozen in liquid nitrogen and stored at −80 °C.

### Body weight

Body weights of rats raised in SL and NL were monitored on a weekly basis. Weighing was performed between the hours of 8 am and 9 am.

### Biochemical analysis

Serum glucose content was determined using a glucose oxidase assay (Rongsheng, Shanghai, China). Levels of triglycerides, high-density lipoprotein cholesterol (HDL-C) and free fatty acids (FFA) were measured using a HITACHI 7180 analyzer with enzymatic reagents (Wako, Osaka, Japan). Serum insulin and leptin content were determined using radioimmunoassay kits (Millipore, Billerica, MA, USA) according to the manufacturer’s instructions. The homeostatic model assessment-insulin resistance (HOMA-IR) index was calculated as follows:

$$ \mathrm{HOMA}-\mathrm{I}\mathrm{R}=\left[\mathrm{fasting}\ \mathrm{serum}\ \mathrm{glucose}\left(\mathrm{mmol}/\mathrm{L}\right)\right]\times \left[\mathrm{fasting}\ \mathrm{serum}\ \mathrm{insulin}\left(\mathrm{mU}/\mathrm{L}\right)\right]/22.5 $$

### Intraperitoneal glucose tolerance test measurement

The intraperitoneal glucose tolerance test (IPGTT) was performed on rats aged 6 weeks and 14 weeks. Rats were fasted overnight for 12 h and a fasting blood sample was taken from a tail vein, then the rats were injected intraperitoneally with 2.0 g D-glucose/kg body weight. Thereafter, blood samples were drawn at 15, 30, 60 and 120 min timepoints and glucose levels were measured using a blood glucose monitoring system (One Touch Sure Step, Johnson & Johnson, New Jersey, USA). The area under the curve (AUC) of IPGTT was calculated using the following formula:

$$ \mathrm{A}\mathrm{U}\mathrm{C} = 0.125 \times \mathrm{B}\mathrm{G}0 + 0.25 \times \mathrm{B}\mathrm{G}15 + 0.375 \times \mathrm{B}\mathrm{G}30 + 0.75 \times \mathrm{B}\mathrm{G}60 + 0.5 \times \mathrm{B}\mathrm{G}120 $$

(BGx: the blood glucose level at 0, 15, 30, 60 and 120 min)

### Quantitative real-time polymerase chain reaction (PCR)

Quantitative real-time PCR was carried out as previous reported [65]. Total RNA was isolated from epididymal fat and gastrocnemius muscles using TRIzol reagent (Invitrogen, USA) according to the kit protocol. The fragmented RNA was reverse-tanscribed into cDNA with the SuperScript double-stranded cDNA synthesis kit (Invitrogen, USA). Primers for amplification of insulin receptor substrate 1 (*Irs-1*), protein kinase B (*Akt2*), glucose transporter 4 (*Glut4*) and the loading control *GAPDH* were designed using Primer Express Software 5.0 (ABI, USA) (Table 5). Real-time PCR was performed on an ABI 7500 instrument (ABI, USA) with a total of 20 μL reaction volumes containing 10 μL of 2X SYBR Green Mix (Invitrogen, USA), 1 μL of cDNA and 0.8 μL of paired specific primers. The reactions were incubated at 50 °C for 2 min and 95 °C for 5 min, followed by 39 cycles of amplification (95 °C for 15 s, 60 °C for 31 s). They were then ramped from 60 °C to 95 °C to obtain the melting curve. Relative mRNA levels were calculated according to the comparative 2^-∆∆Ct^ method [66] and expressed as the fold change compared to the age-matched NL group.

**Table 5:** Primers for gene expression analysis

Genes	Forward primer (5’ → 3’)
	Reversed primer (5’ → 3’)
*Irs-1*	GTGCCAAGCAACAAGAAAGC
	TCAGAGCAGAGGAACCGTAT
*Akt2*	CCACGACCCAACACCTTT
	CCTTGCTGACCGCTACCT
*Glut4 (Slc2a4)*	GGCTGTGAGTGAGTGCTTT
	GGTTTCTGCTCCCTATCGT
*Gapdh*	GGCACAGTCAAGGCTGAGAATG
	ATGGTGGTGAAGACGCCAGTA

### Protein preparation and western blotting

Frozen tissue samples were weighed and homogenized in lysis buffer with protease inhibitor cocktail on ice using a Retsch MM301 mixer mill (Retsch GmbH, Germany). After centrifugation (13000 g) at 4 °C for 1 h, supernatants were transferred to fresh tubes. Protein concentrations in the final supernatants were determined using the bicinchoninic acid (BCA) method (Beyotime Biotechnology, China). For each sample, 50 μg protein was diluted in 2X SDS-PAGE loading buffer and loaded on a 10 % polyacrylamide gel. Proteins were transferred from the gel to a 0.22 μM PVDF membrane using a membrane transfer apparatus (Wealtec, USA). PVDF membranes containing proteins were then rinsed with 1X TBST buffer and blocked in 5 % non-fat milk liquid for 2 h. The blocked membrane was placed in a hybridization bag, and primary antibodies anti-IRS1 (Cell Signaling Technology, USA), anti-AKT2 (Cell Signaling Technology, USA), anti-GLUT4 (Santa Cruz Biotechnology, USA) and anti-GAPDH (Solarbio, China) were added in 5 % milk at 1:500, 1:500, 1:200 and 1:500 dilutions, respectively. Membranes were then rinsed and incubated in goat anti-rabbit-HRP secondary antibody (Beyotime Biotechnology, China), diluted 1:10000 in 5 % milk. The signal was detected by SuperSignal West Pico Chemiluminescent Substrate (Thermo scientific, USA). The results were analyzed using Image J software and normalized to GAPDH.

### Serum fatty acid metabolomics

Acid-catalyzed methyl esterification was used to obtain organic phase samples for fatty acid content of serum sample. BF_3_-methanol (14 %, 1 ml) and internal lipid standard C19:0 (1 mg/ml, 10 μl) were added to every 50 μl serum sample and incubated at 80 °C for 10 min. Water (1 ml) and hexane (1 ml) were added and the samples were vortex mixed for 1 min, then centrifuged at 6000 g for 1 min. The upper (organic) layer was collected for GC-MS analysis.

All GC-MS analyses were performed using a 7000B Triple Quadrupole GC-MS System (Agilent, CA, USA). The organic samples were injected with a split ratio of 10:1 onto a 30 m x 0.25 mm VF-WAXms column (Agilent, CA, USA). The injector temperature was 250 °C and the helium carrier gas was used at a flow rate of 1 ml/min. The column temperature was held at 180 °C for 1 min, then increased by 3 °C/min to 240 °C and held at 240 °C for 10 min [ion source = 280 °C; electron ionization (EI) = 70 eV].

Each individual peak was integrated and normalized (Agilent Mass Hunter Software, Aglient, CA, USA). Overlapping peaks were separated using traces of single ions. Peak assignment was based on mass fragmentation patterns matched to the National Institute of Standards and Technology library, and a fatty acid methyl ester (FAME) standard mix (Supelco 37 component FAME mix, Sigma-Aldrich, Shanghai, China).

### Statistical analysis

Results are expressed as mean ± S.E.M. Body weights and IPGTTs were analyzed by two-way ANOVA with repeated measures followed by a *post hoc* least significance difference test (SPSS 17.0). Organ weights, serum biochemistry, serum fatty acid profile, serum leptin and insulin concentrations, HOMA-IR, mRNA value and western blot results were analyzed using Student’s *t-*test. Correlation coefficients between HOMA-IR and individual serum fatty acids were calculated according to the Pearson correlation test. Significant differences were defined as *P* < 0.05.

## Abbreviations

IR: Insulin resistance
NL: Normal litter
SL: Small litter
IRS: Insulin receptor substrate
Akt2: Protein kinase B
GLUT4: Glucose transporter 4
FFA: Free fatty acid
IPGTT: Intraperitoneal glucose tolerance test
AUC: Area under the curve
HOMA-IR: Homeostatic model assessment-insulin resistance
HDL-C: High density lipoprotein-cholesterol
SFAs: Saturated fatty acids
MUFAs: Monounsaturated fatty acids
PUFAs: Polyunsaturated fatty acids
